# BioModTool: from biomass composition data to structured biomass objective functions for genome-scale metabolic models

**DOI:** 10.1093/bioadv/vbaf036

**Published:** 2025-02-21

**Authors:** Clémence Dupont Thibert, Sylvaine Roy, Gilles Curien, Maxime Durot

**Affiliations:** Laboratoire de Physiologie Cellulaire et Végétale, Interdisciplinary Research Institute of Grenoble, Université Grenoble Alpes, Grenoble 38000, France; Centre de Recherche de Solaize, TotalEnergies OneTech, Solaize 69360, France; Laboratoire de Physiologie Cellulaire et Végétale, Interdisciplinary Research Institute of Grenoble, Université Grenoble Alpes, Grenoble 38000, France; Laboratoire de Physiologie Cellulaire et Végétale, Interdisciplinary Research Institute of Grenoble, Université Grenoble Alpes, Grenoble 38000, France; Centre de Recherche de Solaize, TotalEnergies OneTech, Solaize 69360, France

## Abstract

**Summary:**

BioModTool is a Python program allowing easy generation of biomass objective functions for genome-scale metabolic models from user data. BioModTool loads biomass composition data in the form of a structured Excel file completed by the user, normalizes these data into model-compatible units (mmol.gDW^−1^), and creates a structured biomass objective function to update a metabolic model. Aimed at a wide range of users, BioModTool can be run as a Python module compatible with COBRApy but also comes with an interface allowing its use by non-modelers. By providing an easy definition of new biomass objective functions, BioModTool can accelerate new genome-scale metabolic reconstructions, improve existing ones, and facilitate biomass-specific experimental datasets analyses with genome-scale models.

**Availability and implementation:**

BioModTool is publicly available on PyPI (https://pypi.org/project/BioModTool/) under a GNU Lesser General Public License (LGPL). Installation instructions and source code are available on GitHub (https://github.com/Total-RD/BioModTool). BioModTool is compatible with Windows, Linux, and MacOS operating systems.

## 1 Introduction

A genome-scale metabolic model (GEM) is a computational network representing all known enzymatic and spontaneous metabolic reactions and metabolic genes of a given organism. A crucial step in GEM reconstruction is the definition of biomass reactions, commonly referred to as Biomass Objective Function (BOF) (Feist and Palsson 2010). A BOF consumes all metabolites required to produce 1 g of dry-weight (gDW) biomass and is widely used as simulation objective using Constraint-Based Reconstruction and Analysis (COBRA) methods such as Flux Balance Analysis (FBA) (Ebrahim *et al.* 2013, O’Brien *et al.* 2015, Gu *et al.* 2019, Heirendt *et al.* 2019). GEMs usually assume a constant biomass composition, while *in vivo* biomass composition is adjusted in response to changing environmental conditions: nutrient availability, temperature, stress, etc. (Schaechter *et al.* 1958, Cotner *et al.* 2006, Zuñiga *et al.* 2018). Moreover, numerous studies have demonstrated the high impact of biomass composition on model behaviors and predictions (Pramanik and Keasling 1998, Nookaew *et al.* 2008, Lavoie *et al.* 2020, Schulz *et al.* 2021). High-quality, condition-dependent BOFs are therefore necessary to enable reliable predictions in GEMs.

The need of accurate characterization of the biomass composition has been addressed over the years by experimental protocols (Feist and Palsson 2010, Beck *et al.* 2018, Simensen *et al.* 2022). Converting experimental data into BOF stoichiometric coefficients is fastidious and requires several normalization and unit conversion steps, which can be source of errors. Despite the importance of BOF in GEMs, there is a significant lack of computational tools to generate species- or condition-specific BOFs from data (Xavier *et al.* 2017, Beck *et al.* 2018, Lachance *et al.* 2019, von Kamp and Klamt 2023). During model reconstruction, modelers often adopt the BOF from an established high-quality GEM, rather than creating one from scratch. Reconstruction tools such as SEED and CarveMe facilitate this process by providing pre-defined BOFs for selection based on the phylogeny of the organism (Devoid *et al.* 2013, Machado *et al.* 2018). Some tools using fluxomics data to predict the metabolic end goals of the cell are also available, including ObjFind, BOSS, or invFBA (Burgard and Maranas 2003, Gianchandani *et al.* 2008, Zhao *et al.* 2016). Nevertheless, this approach is currently limited by the availability of fluxomic data. Recently, BOFdat, a Python workflow computing BOF from different datasets including genomic, transcriptomic, proteomic, and lipidomic was developed (Lachance *et al.* 2019). However, format-specific multi-omics data are rarely available, therefore limiting the number of organisms and conditions in which BOFdat can be applied.

Here, we present BioModTool (Biomass Modeling Tool), a new Python package for the complete definition of organism- or condition-specific BOF of an existing GEM from a single editable structured dataset. Being provided with template Excel files (Supplementary Files S1–S3) to be filled by the user with biomass composition data, BioModTool offers great flexibility in terms of data sources, formats, and units. Thus, biomass composition data can be found in the literature, determined using appropriate protocols or even determined using the appropriate BOFdat function when a specific omic dataset is available. First, BioModTool calculates stoichiometric coefficients of all metabolites to be consumed in BOF, involving normalization and unit conversion steps. Pseudo-reactions and pseudo-metabolites required for BOF definition are then created and added to the GEM to update.

## 2 Methods

### 2.1 BOF structure

**Figure 1.**
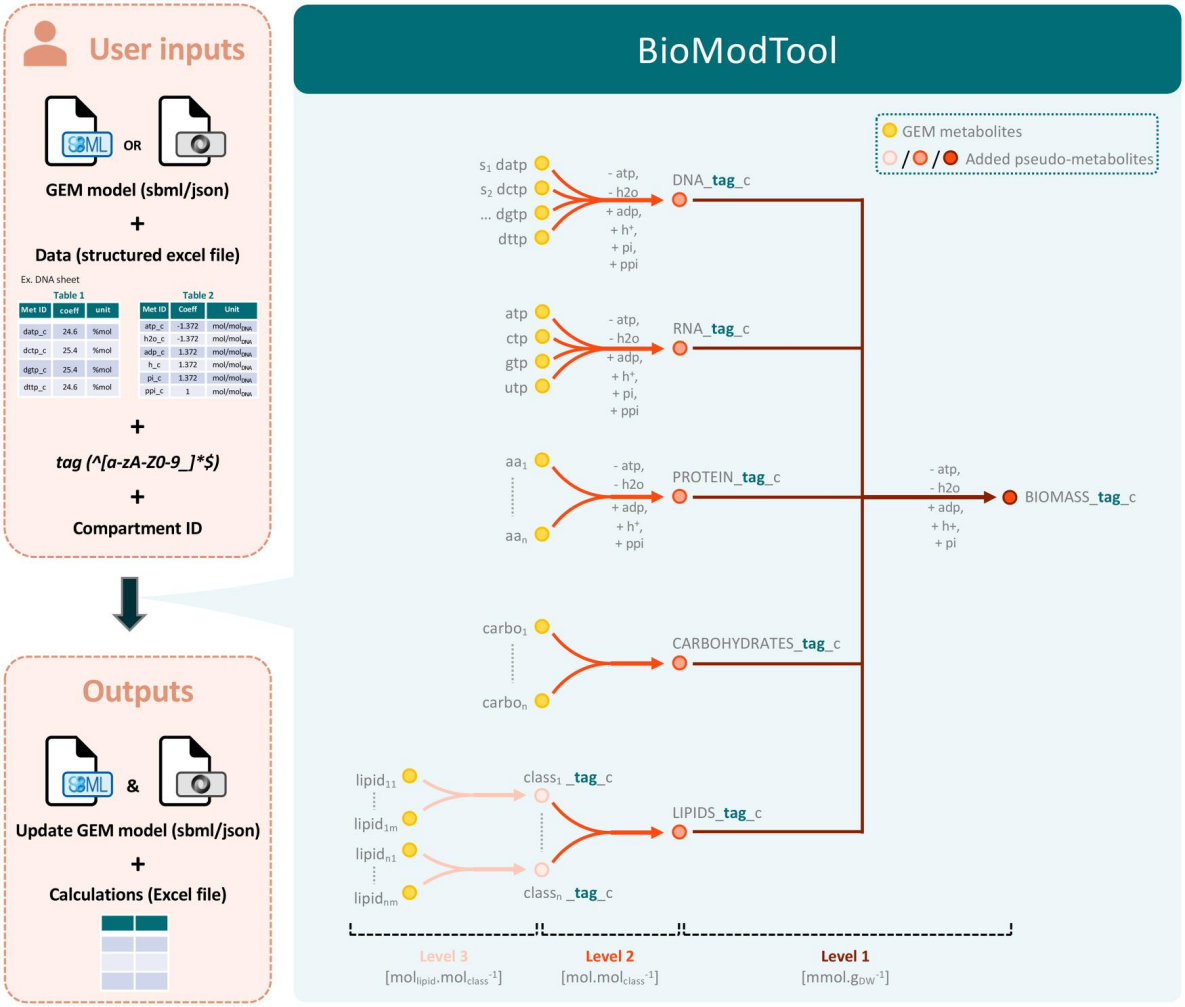
BioModTool usage to generate and add a new BOF to GEM. First, BioModTool loads and tests user inputs (GEM, data, tag, and compartment identifier). Then, biomass composition data are normalized and converted in appropriate units, respecting mass balance. Pseudo-metabolites and pseudo reactions are instantiated and added to the GEM. When possible, formula and charges are calculated and added to pseudo-metabolite attributes. Stoichiometric coefficient calculations results and updated GEM are, respectively, saved as XLSX and SBML/JSON files. Circles indicate metabolites existing in the chosen GEM (yellow) and pseudo-metabolites that will be added to GEM by BioModTool (reddish). Arrows represent pseudo-reactions created and added by BioModTool. Final stoichiometric coefficients units of the three levels are indicated between brackets.

Before running BioModTool, the user must define the BOF structure. BOFs are commonly organized into different levels, each level being described by appropriate pseudo-reaction(s). Up to three BOF levels can be defined by BioModTool (Fig. 1). The first level consumes the major macromolecules of the cell such as DNA, RNA, proteins, carbohydrates, lipids, etc., and is typically defined as follows:

$$s_{1}{\;\text{DNA}+s}_{2}{\;\text{RNA}+s}_{3}{\;\text{PROTEINS}+s}_{4}{\;\text{CARBOHYDRATES}+s}_{5}\;\text{LIPIDS}+\ldots \;\to \;1\;\text{BIOMASS}\;(\text{Level}\;1)$$

where s_i_ are the calculated macromolecules abundance in cell biomass (in mmol.gDW^−1^.h^−1^) and DNA, RNA, PROTEINS, etc. are pseudo-metabolites resulting from the consumption of appropriate monomers [such as deoxynucleotide triphosphates (dNTP), amino acids etc.]. For example, DNA is a pseudo-metabolite produced by a pseudo-reaction consuming the four dNTPs:

$$s_{1}{\;\text{dATP}+s}_{2}\;\text{dCTP}+\ldots \;\to 1\;\text{DNA}\;(\text{Level}\;2)$$

where s_i_ is the molar fraction of each metabolite in the macromolecule (here in mol.${\text{mol}}_{\text{DNA}}^{-1}$).

Due to the diversity of existing lipid entities, lipid pseudo-reactions are usually more challenging to formulate. One solution consists in introducing additional pseudo-reactions and pseudo-metabolites to gather lipid metabolites by class. This is possible with BioModTool by introducing a third level in the BOF. Lipid class consideration requires definition of lipid pseudo-reaction as follows:

$$s_{1}{\;\text{PC}+s}_{2}\;\text{PE}+\ldots \;\to \;1\;\text{LIPIDS}\;(\text{Level}\;2-\text{lipid})$$

where s_i_ is the molar fraction of the lipid class in total lipids (mol_class_.${\text{mol}}_{\text{LIPIDS}}^{-1}$) and PC (phosphatidylcholine), PE (phosphatidylethanolamine), etc. are pseudo-metabolites representing each lipid class described to be consumed in the BOF. Lipid class consideration can consequently result in the addition of a third level of pseudo-reactions, consuming specific lipids. For example, for PC:

$$s_{1}{\;\text{PC}(14\text{:}0/14\text{:}0)+s}_{2}\;\text{PC}(16\text{:}0/16\text{:}0)+\ldots \;\to \;1\;\text{PC}\;(\text{Level}\;3)$$

where s_i_ is the molar fraction of each specific lipid in its given class (here in mol.${\text{mol}}_{\text{PC}}^{-1}$).

Levels 2 and 3 are optional, and a single level BOF can be generated by BioModTool. The aforementioned macromolecules are just examples. Additional macromolecules, such as pigments for photosynthetic organisms, can be added at the user’s convenience. The same applies to lipid classes. For each pseudo-reaction, maintenance costs of growth or pseudo-metabolite synthesis, the requirement of necessary vitamins, elements, and cofactors, etc. can also be included in the BOF generated by BioModTool.

### 2.2 Biomass composition

User must update and complete one of the provided Excel files (single-level BOF, two-level BOF, three-level BOF; Supplementary Files S1–S3) with biomass composition data and metabolite identifiers of the chosen GEM according to the desired BOF structure. Excel file is divided into sheets, each sheet corresponding to a pseudo-reaction to be created (e.g. BIOMASS, DNA, LIPIDS, PC, etc.). Each sheet contains two distinct tables. The first table (columns A–E) contains metabolites of basic level defining the macromolecular or monomer content in the cell. The units used can vary between the pseudo-reactions to be created (one Excel sheet corresponding to one pseudo-reaction), but the metabolites consumed in a given pseudo-reaction must be given in the same unit. For example, DNA and PROTEIN composition can be given in different units, but all amino-acid abundance must be in the same unit (idem for dNTPs). These data will be normalized so that the total mass recovered from all data corresponds to 1 g of dry weight before being converted into GEM-compatible units.

BOF can be formulated at a different level of detail (basic, intermediate, and advanced) (Feist and Palsson 2010). A second table (columns H–L) can be completed by the user to improve BOF level of details by adding energy requirement for the macromolecule synthesis (intermediate level) and vitamins, elements, and cofactors required for growth (advanced level). Data Table 2 will not be converted and will directly be used as stoichiometric coefficients; therefore, they must be given in mmol.gDW^−1^ or mol.${\text{mol}}_{\text{pool}}^{-1}$. In BioModTool, all polymers are considered to be one monomer long, stoichiometric coefficients of energy and byproducts requirements must be given on the basis of these polymer lengths.

Details on defining the structure and composition of the BOFs are given in Supplemental_Text1.

### 2.3 Run BioModTool

BioModTool can be run either in Python or using the user interface (implemented with Tkinter). In both cases, user must provide several inputs (Fig. 1).

1. A GEM (in SBML or JSON format) containing at least one compartment with an ID. According to the chosen model, user must indicate if charge and/or formula are available for all metabolites consumed in the BOF and must define in which compartment the BOF will be added (among compartments available in model).
2. The Excel file as described above, filled with biomass composition data and metabolites identifiers. Metabolites identifiers must comply with the chosen GEM identifiers.
3. A string defining a “tag” (unique identifier) that will be added as a suffix to all pseudo-metabolites and pseudo-reactions of the newly generated BOF. This “tag” facilitates identification of all pseudo-reactions and pseudo-metabolite constituting a BOF and provides the possibility to introduce several BOFs in a single GEM.

A function to remove a BOF given its tag is also available in BioModTool (not in the graphical interface version).

Installation and usage procedures are fully detailed in Supplemental_Text1 and are available on GitHub (https://github.com/Total-RD/BioModTool/tree/main/BioModTool_Documentation).

## 3 Results

To demonstrate its applicability, BioModTool was applied to two bacteria GEMs—*Escherichia coli* (iML1515; Monk *et al.* 2017) and *Alicyclobacillus acidocaldarius* [CNA_Alicyclo (Beck *et al.* 2018)] —and one microalga *Chlamydomonas reinhardtii* (iRC1080; Chang *et al.* 2011), using data provided in Beck *et al.* (2018) for iML1515 and CNA_Alicyclo, and in Chang *et al.* (2011) for iRC1080. Generated BOFs were validated in terms of charge and mass balance. Stoichiometric coefficients and calculated formula and charges were also validated by comparison with expected values (Supplemental_Text2). For each updated GEM, its ability to predict growth by parsimonious FBA using the newly added BOF as objective was tested and validated. Predicted growth rates were consistent with results obtained using original BOF. The scripts and required input files for these examples are available on GitHub (https://github.com/Total-RD/BioModTool/tree/main/Application_examples).

## 4 Conclusion

BioModTool bridges the gap between biomass composition data and generation of structured BOFs. By allowing easy creation of new BOFs, it provides a valuable tool for GEM reconstruction and refinement, and will be useful to account for the dependence of biomass composition on environmental conditions in GEM. Unlike other available tools, restricted to experienced modelers and currently limited by the availability of omics datasets in specific formats, BioModTool offers great flexibility. It offers flexibility not only in usable sources to obtain biomass composition data (literature, experimental measurements, utilization of other computational tools such as BOFdat functions with a given omic dataset, etc.) but also in level of details that can be included in BOF, ranging from basic level to advanced level depending on data availability. This flexibility makes BioModTool usable in many situations. Finally, availability of a graphical interface makes BioModTool the only biomass reconstructing tool usable by non-modelers. When combined to existing interactive simulation tools, such as ESCHER-FBA (Rowe *et al.* 2018), BioModTool has the potential to further spread GEM applications.

## Supplementary Material

vbaf036_Supplementary_Data

## Data Availability

BioModTool is implemented in Python and is publicly available on PyPI (https://pypi.org/project/BioModTool/) under a GNU Lesser General Public License (LGPL). The source code of the BioModTool, the interface script and executable, as well as the scripts and data used for validation are available on GitHub (https://github.com/Total-RD/BioModTool).
