# Supplementary material for: BioModTool: from biomass composition data to structured biomass objective functions for genome-scale metabolic models
Source: Bioinform Adv. 2025 Feb 21;5(1):vbaf036. doi: 10.1093/bioadv/vbaf036 (PMC11891441; doi:10.1093/bioadv/vbaf036)
Supplement: vbaf036_Supplementary_Data [file vbaf036_supplementary_data.zip › 20240617_Supp_Text_2_Application_Examples.docx]

**BioModTool – Validation**

BioModTool was applied to GEM of two bacteria species *Escherichia coli* (iML1515, (Monk et al. 2017)) and *Alicyclobacillus acidocaldarius* (CNA_Alicyclo, (Beck, Hunt et Carlson 2018)), and one microalga *Chlamydomonas reinhardtii* (iRC1080, (Chang et al. 2011)). These examples illustrate the three different contexts in which BioModTool can be used:

- Add a three level BOF in a model in which metabolic formula are available: iML1515
- Add a two level BOF in a model in which metabolic formula are not available: CNA_Alicyclo
- Add a one level BOF in a model in which metabolic formula are not available: iRC1080

All scripts and results of these three application examples are fully available: <https://github.com/Total-RD/BioModTool/tree/main/Application_examples>.

1. Addition of a 3 levels BOF to iML1515 GEM
   1. Methods
      1. BOF addition using BioModTool

BioModTool was applied to iML1515, the latest version of the *E. Coli* model (Monk et al. 2017). Biomass composition data were obtained from (Beck, Hunt et Carlson 2018) (File S4_Biomass_Composition). Data available allow definition of a three levels BOF. Level 1 pseudo-reaction is defined as:

s_1_ DNA + s_2_ RNA + s_3_ PROTEINS + s_4_ LIPIDS + s_5_ POLYSACCHARIDES 🡪 1 BIOMASS (Level 1)

With s_i_ stoichiometric coefficients. Level 2 is composed of five pseudo-reactions, one for each aforementioned macromolecules, with lipid pseudo-reaction defined as:

s_6_ PE + s_7_ PG + s_8_ CLPN 🡪 1 LIPIDS (Level 2 - lipids)

Finally, level 3 is defined by three pseudo-reactions, one for each lipid class.

Raw data were used to fill BioModTool Excel (Supplemental File S4). BioModTool was used to generate and add a new BOF to iML1515 with following parameters: dict_structure = {'POLYSACCHARIDES': 'level_2', 'DNA': 'level_2', 'RNA': 'level_2', 'PROTEINS': 'level_2', 'LIPIDS': 'level_2_lipid', 'PG': "level_3", 'PE': "level_3", 'CLPN': "level_3", 'BIOMASS': 'level_1'}, calculate_charge = True, calculate_formula = True.

- - 1. Addition of Beck BOF to iML1515

Beck et al. 2018 introduce a BOF for *E. Coli*, defined by six reactions (Table 1). These reactions were added to iML1515. Macromolecule fraction stoichiometric coefficients are expressed in mol_macromolecule_/100kg_DW._ These coefficients were converted in mmol_macromolecule_.g_DW_^-1^ to allow comparison with BioModTool BOF. Moreover, in Beck et al. 2018 polymer lengths for the macromolecular synthesis reactions was set to 10 NTPs for RNA, 10 glucose-1-phosphates for polysaccharides and 100 amino acids for proteins. Stoichiometric coefficients were adjusted so that all polymers are one monomer long.

Table 1. Beck BOF reactions.

| **Reaction ID** | **Equation** |
| --- | --- |
| Biomass reaction | 5.05029378916591 DNA + 8.40374146379437 RNA + 5.01641118949985 protein + 13.9019957911911 lipid + 4.02851950095506 polysaccharide = 1 biomass |
| DNA synthesis reaction | 0.246 dATP + 0.254 dCTP + 0.254 dGTP + 0.246 dTTP = 1 DNA + 1 diphosphate |
| RNA synthesis reaction | 2.0262150129207 ATP + 3.1523397366045 CTP + 2.25131542077898 GTP + 2.57012982969582 UTP = 1 RNA + 10 diphosphate |
| Protein synthesis reaction | 10.1 Alanine + 1.2 Cysteine + 5.38333333333333 Aspartate + 6.88039215686274 Glutamate + 3.9 Phenylalanine + 10.9 Glycine + 1.6 Histidine + 4.8 Isoleucine + 5.8 Lysine + 7.2 Leucine + 2.8 Methionine + 4.11666666666667 Asparagine + 4 Proline + 5.21960784313726 Glutamine + 5 Arginine + 4.9 Serine + 5.3 Threonine + 6.8 Valine + 1.5 Tryptophan + 2.8 Tyrosine + 100 ATP + 198 GTP + 197.8 H2O = 1 protein + 100 AMP + 100 diphosphate + 198 GDP + 198 phosphate + 298 H+ |
| Lipid synthesis reaction | 0.76530612244898 Phosphatidylethanolamine + 0.183673469387755 Phosphatidylglycerol + 0.0510204081632653 Cardiolipin = 1 lipid |
| Polysaccharide synthesis reaction | 10 Glucose-1-phosphate + 10 ATP = 1 polysaccharide + 10 ADP + 10 diphosphate |

- - 1. Predict maximum growth rate using pFBA

Flux distributions were performed using open-source COBRApy package (Ebrahim et al. 2013); version 0.26.3), with Python version 3.11.4. Maximum growth rate was predicted by Parsimonious Flux Balance Analysis (pFBA) with respectively original Beck BOF and BioModTool_Ecoli BOF as objective functions. Default iML1515 constrains were kept unchanged for these simulations.

- 1. Results

BioModTool was used to add the generate a new BOF for *E. coli* from (Beck, Hunt et Carlson 2018) biomass composition data. Final coefficients, pseudo-metabolites formula and charge calculated by BioModTool were compared to (Beck, Hunt et Carlson 2018) calculations (Table 1). Maximum growth rate was predicted using pFBA with respectively Beck BOF and BioModTool_Ecoli BOF as objective functions. A maximum growth rate of 1.1301 h^-1^ was predicted for both BOFs. All reactions constituting BioModTool_Ecoli BOF are mass and charge balanced.

Table 2. Comparison of BioModTool calculations to Beck et al., 2018 for *E. coli*.

|  | **Beck et al., 2018 calculations** | | | | **BioModTool calculations** | | |
| --- | --- | --- | --- | --- | --- | --- | --- |
|  | **Coefficient** [mol polymer/  100 kg cdw] | **Coefficient** [mmol.gDW-1] | **Formula** | **Charge** | **Coefficient** [mmol.gDW-1] | **Formula** | **Charge **** |
| DNA | 5.05 | 5.05E-02 | C_9.75_H_11.25_N_3.75_O_6_P_1_ | -1 | 5.05E-02 | C_9.746_H_11.246_O_6.0_N_3.754_P_1.0_ | -1 |
| RNA * | 8.40 | 8.40E-02 | C_94.28_H_107.43_N_35.98_O_70.54_P_10_ | -10 | 8.40E-01 | C_9.428_H_10.743_O_7.0543_N_3.598_P_0.1_ | -1 |
| protein * | 5.02 | 5.02E-02 | C_481.30_H_753.37_N_135.04_O_147.06_S_4_ | -1.46 | 5.03 | C_4.803_H_7.514_O_1.466_N_1.348_S_0.04_ | 0 |
| lipid | 1.39E+01 | 1.39E-01 | C_40.99_H_78.73_N_0.18_O_10.01_P_1.05_ | -2.86E-01 | 1.40E-01 | C_40.061_H_78.074_O_8.826_N_0.765_P_1.051_ | 0 |
| polysaccharide * | 4.03 | 4.03E-02 | C_60_H_100_O_50_ | 0 | 4.03E-01 | C_6.0_H_10.0_O_5.0_ | 0 |
| **Biomass formula** | |  | C_4059_H_6236_N_1009_O_1685_P_104_S_20_ | |  | C_40.593_H_62.339_O_16.851_N_10.097_P_1.038_S_0.201_ | |
| **Biomass formula normalized to C** | | | CH_1.54_N_0.25_O_0.42_P_0.03_S_0.005_ |  |  | C_1_H_1.54_O_0.42_N_0.25_P_0.03_S_0.005_ |  |

** In (Beck et al. 2018) polymer lengths for the macromolecular synthesis reactions was set to 10 NTPs for RNA, 10 glucose-1-phosphates for polysaccharides and 100 amino acids for proteins. On the other hand, in BioModTool all polymers are considered to be one monomer long. This difference explains the observed factor of 10 and 100 between the results of Beck and the results of BioModTool for DNA and polysaccharide, and proteins respectively. ** In GEM models, charge must be an integer. In BioModTool charges are therefore rounded to the nearest integer value.*

1. Addition of 2 levels BOF to CNA_Alicyclo *A.* *acidocaldarius* GEM
   1. Methods
      1. BOF addition using BioModTool

BioModTool was applied to CNA_Alicyclo model (Beck, Hunt et Carlson 2018). Biomass composition data were obtained from (Beck, Hunt et Carlson 2018) (File S4_Biomass_Composition). Data available allow definition of a two levels BOF. Level 1 pseudo-reaction is defined as:

s_1_ DNA + s_2_ RNA + s_3_ PROTEINS + s_4_ LIPIDS + s_5_ POLYSACCHARIDES 🡪 1 BIOMASS (Level 1)

Level 2 is composed of five pseudo-reactions, one for each aforementioned macromolecules.

Raw data were used to fill BioModTool Excel (Supplemental File S5). Since no formula are available in CNA_Alicyclo, no data conversion, requiring molecular weight which is calculated from metabolite formula, can be performed by BioModTool. Biomass macromolecular composition was therefore given in final unit: mmol.gDW^-1^. BioModTool was used to generate and add a new BOF to CNA_Alicyclo (Beck, Hunt et Carlson 2018) with following parameters: dict_structure = {'POLYSACCHARIDES': 'level_2', 'DNA': 'level_2', 'RNA': 'level_2', 'PROTEINS': 'level_2', 'LIPIDS': 'level_2', 'BIOMASS': 'level_1'}, calculate_charge = False, calculate_formula = False.

- - 1. Predict maximum growth rate using pFBA

Flux distributions were performed using open-source COBRApy package (Ebrahim et al. 2013) version 0.26.3), with Python version 3.11.4. Maximum growth rate was predicted by Parsimonious Flux Balance Analysis (pFBA) with respectively original B0 BOF and BioModTool_Aacidocaldarius BOF as objective functions. Default model constrains were kept unchanged for these simulations.

- 1. Results

BioModTool was used to add the generate a new BOF for *A.* *acidocaldarius* from (Beck, Hunt et Carlson 2018) biomass composition data. BioModTool_Aacidocaldarius BOF coefficients were compared to (Beck, Hunt et Carlson 2018) calculations (Table 3). Maximum growth rate was predicted using pFBA with respectively original B0 BOF and BioModTool_Aacidocaldarius BOF as objective functions. A maximum growth rate of 35.842 h^-1^ was predicted for both BOFs.

Table 3. Comparison of BioModTool coefficients to Beck et al., 2018 for *A.* *acidocaldarius.*

|  | **Beck et al., 2018** | | **BioModTool** |
| --- | --- | --- | --- |
|  | **Coefficient** [mol polymer/100 kg cdw] | **Coefficient** [mmol.gDW-1] | **Coefficient** [mmol.gDW-1] |
| DNA | 3.49E+00 | 3.49E-02 | 3.49E-02 |
| RNA * | 7.91E+00 | 7.91E-02 | 7.91E-01 |
| Protein * | 5.46E+00 | 5.46E-02 | 5.46 |
| lipid | 6.67E+00 | 6.67E-02 | 6.67E-02 |
| Polysaccharides * | 5.86E+00 | 5.86E-02 | 5.86E-01 |

** In (Beck et al. 2018) polymer lengths for the macromolecular synthesis reactions was set to 10 NTPs for RNA, 10 glucose-1-phosphates for polysaccharides and 100 amino acids for proteins. On the other hand, in BioModTool all polymers are considered to be one monomer long. This difference explains the observed factor of 10 and 100 between the results of Beck and the results of BioModTool for DNA and polysaccharide, and proteins respectively.*

1. Addition of single level BOF to iRC1080 *C. reinhardtii* GEM
   1. Methods
      1. BOF addition using BioModTool

BioModTool was applied to iRC1080 model of *Chlamydomonas* *reinhardtii* (Chang et al. 2011). Biomass composition data were obtained from (Chang et al. 2011) (msb201152-s13.xlsx file, Autotrophic data). Data available allow definition of a BOF with only one level. BOF reaction is defined as:

s_1_ dttp_c + s_2_ utp_c + s_3_ gly_c+ s_4_ mgdg1829Z12Z1617Z_h+ s_5_ but_c … 🡪 1 BIOMASS (Level 1)

Since formula is missing for several metabolites consumed in BOF reaction, no data conversion (requiring molecular weight which is calculated from metabolite formula) can be performed by BioModTool. All coefficients were therefore given in mmol.gDW^-1^mmol.gDW^-1^ in BioModTool Excel (Supplemental File S6). BioModTool was used to generate and add the new BOF to iRC1080 with following parameters: dict_structure = {''BIOMASS': 'level_1'}, calculate_charge = False, calculate_formula = False.

- - 1. Predict maximum growth rate using pFBA

Flux distributions were performed using open-source COBRApy package (Ebrahim et al. 2013); version 0.26.3), with Python version 3.11.4. Maximum growth rate was predicted by Parsimonious Flux Balance Analysis (pFBA) with respectively original Biomass_Chlamy_auto BOF and BioModTool_Creinhardtii BOF as objective functions. Default model constrains were kept unchanged for these simulations.

- - 1. Results

Maximum growth rate was predicted using pFBA with respectively original Biomass_Chlamy_auto BOF and BioModTool BOF as objective functions. A maximum growth rate of 6.157 h^-1^ was predicted for both BOFs.

1. Conclusion

Taken together, these three application examples validate the efficiency of BioModTool in generating functional BOF from user data. These examples also underscore the flexibility of BioModTool in terms of (1) updatable GEM, (2) generated BOFs structure (with one, two, or three levels) and (3) BOF complexity including basic, intermediate and advanced levels metabolites.

References

Beck, Ashley; Hunt, Kristopher; Carlson, Ross (2018): Measuring Cellular Biomass Composition for Computational Biology Applications. In Processes 6 (5), p. 38. DOI: 10.3390/pr6050038.

Chang, Roger L.; Ghamsari, Lila; Manichaikul, Ani; Hom, Erik F. Y.; Balaji, Santhanam; Fu, Weiqi et al. (2011): Metabolic network reconstruction of Chlamydomonas offers insight into light-driven algal metabolism. In Molecular systems biology 7, p. 518. DOI: 10.1038/msb.2011.52.

Monk, Jonathan M.; Lloyd, Colton J.; Brunk, Elizabeth; Mih, Nathan; Sastry, Anand; King, Zachary et al. (2017): iML1515, a knowledgebase that computes Escherichia coli traits. In Nature biotechnology 35 (10), pp. 904–908. DOI: 10.1038/nbt.3956.

## 
