# Supplementary material for: BioModTool: from biomass composition data to structured biomass objective functions for genome-scale metabolic models
Source: Bioinform Adv. 2025 Feb 21;5(1):vbaf036. doi: 10.1093/bioadv/vbaf036 (PMC11891441; doi:10.1093/bioadv/vbaf036)
Supplement: vbaf036_Supplementary_Data [file vbaf036_supplementary_data.zip › 20250113_Supp_Text_1_Installation_and_Usage.docx]

BioModTool – Install and Usage

1. BioModTool installation

Use pip to install BioModTool from PyPI (<https://pypi.org/project/BioModTool/>).

*pip install BioModTool*

BioModTool source code can also be downloaded or cloned from GitHub (<https://github.com/Total-RD/BioModTool/tree/main/BioModTool>). You can run the following to install BioModTool.

*pip install <path-to-BioModTool-repo>*

1. Determine biomass composition and structure

Before using BioModTool, the user must (1) define the composition of the biomass to be modeled and (2) determine the structure of the biomass function to be added to the model. To do this, the user must adapt to the model he's working on as well as to the available data.

- 1. Defining biomass composition

Before using BioModTool, the user must define the composition of the biomass function to be added. BioModTool does not require a specific biomass composition to be used. However, to create a relevant biomass function, certain metabolites must be consumed. Thus, the biomass function should consume all compounds needed to create a new cell, including DNA, amino acids, lipids, and polysaccharides. Biomass functions can also include details of the energy (ATP), vitamins, and cofactors required for growth (Feist and Palsson 2010). Issues related to the formulation of biomass objective functions were reviewed by Feist and Palsson in 2010 (Feist and Palsson 2010).

- - 1. Data sources

BioModTool offers flexibility in terms of the origin and format of usable data.

Users can use their own data or data from the literature to determine biomass composition. In addition, several biomass composition analysis protocols have been developed for GEM reconstruction (Thiele and Palsson 2010; Beck, Hunt and Carlson 2018; Simensen et al. 2022). However, data availability can vary considerably depending on the organism studied or its culture conditions. Comparison of available data with data and/or models from organisms phylogenetically close to the organism of interest may also be useful in determining biomass composition. Independent functions of existing tools such as BOFdat (Lachance 2019) can also be used with appropriate omic data to determine some metabolite stoechiometric coefficients.

- - 1. Data units

There is a real diversity of units for expressing the abundance of a metabolite in an organism (Tolleter et al. 2024). Various units of mass and molar concentration can be used to characterize biomass (Table 1).

Table 1. Examples of units of mass and molar concentration used to describe biomass composition.

| Units of mass concentrations | Units of molar concentration |
| --- | --- |
| - Grams per liter  - Grams per cell  - Grams per gram of dry weight  - Percentage or mass fraction  - Grams per gram of protein (for amino acids)  - Grams per gram of total lipid or grams per gram of TAG (for TAGs)  Etc. | - Moles per liter  - Moles per cell  - Moles per gram of dry weight  - Percentage or mole fraction  - Moles per moles of DNA (for dNTPs)  - Moles per moles of total lipid or moles per moles of TAG (for TAGs)  Etc. |

When using BioModTool, the unit of basic metabolites is selected from a drop-down list. Only two units are proposed to the user: “g per ...” and “mol per ...” respectively representing all the mass and molar concentration units. The data are then normalized converted by BioModTool to respect metabolic modeling constraints. For calculation details see part 4. Implementation.

- 1. Determining the Structure of the Biomass Function

Although it is possible to model a biomass function by a single reaction that consumes all metabolites in the appropriate proportions, it is common practice to represent the biomass function by several pseudo-reactions organized in two or three levels. Thus, the first level consumes the main macromolecules of the cell, such as DNA, RNA, proteins, carbohydrates, lipids, and so on. The second level corresponds to the consumption of monomers (dNTPs, amino acids, etc.), symbolizing the polymerization of macromolecules. A third level can also be added to detail the profile of different lipid classes. The functional composition of biomass can be divided into three levels of detail: (1) basic, (2) intermediate, and (3) advanced (Feist and Palsson 2010). The composition of the biomass function also depends on knowledge of the cellular composition and energy requirements. Initially, GEMs tend to have basic-level functions that consume only the major macromolecules (RNA, DNA, proteins, and lipids). These formulations are then refined and expanded to include energy consumption for nucleic acid and protein polymerization (intermediate level). Finally, metabolites specific to the organism of interest, such as vitamins, cofactors, inorganic ions, and membrane components, can be added (advanced level). BioModTool is compatible with these different levels of detail.

For further information on biomass function structure and composition please refer to by Feist and Palsson review (Feist and Palsson 2010).

1. BioModTool usage

Usage of BioModTool is illustrated using the example of *E. Coli* iML1515 GEM (Monk et al. 2017). Data used to determine *E. Coli* biomass composition come from S4_Biomass_Composition supplementary file from Beck, Hunt et Carlson 2018.

- 1. BOF structure and Biomass composition

(Beck, Hunt et Carlson 2018) provide monomer and macromolecular composition of the biomass for: DNA, RNA, proteins, lipids and polysaccharides. Regarding lipids, three classes are defined: PE (Phosphatidylethanolamine), PG (Phosphatidylglycerol) and CLPN (Cardiolipin). A single fatty acid profile is available. Same fatty acid profile for the three lipid classes was therefore considered. Given available data, BOF is structured as follows (with s_i_ representing stoichiometric coefficients):

s_1_ DNA + s_2_ RNA + s_3_ PROTEINS + s_4_ LIPIDS + s_5_ POLYSACCHARIDES 🡪 1 BIOMASS (Level 1)

Level 2 is composed of five pseudo-reactions, one for each aforementioned macromolecules, with lipid pseudo-reaction defined as:

s_6_ PE + s_7_ PG + s_8_ CLPN 🡪 1 LIPIDS (Level 2 - lipids)

Finally, level 3 is defined by three pseudo-reactions: one for each lipid class.

(Beck, Hunt et Carlson 2018) data were used to complete BioModTool Excel file. For example, "BIOMASS" sheet was filled based on macromolecular/polymer composition of biomass (Table 2).

Table 2. Macromolecular composition of E. Coli (Beck, Hunt et Carlson 2018)

| **Macromolecule** | **g_polymer_.g_DW_^-1^** |
| --- | --- |
| DNA | 1.0 |
| RNA | 17.2 |
| proteins | 35.2 |
| lipids | 6.7 |
| polysaccharides | 4.2 |

Raw data are given in g_polymer_.g_DW_^-1^, so unit "g per …" was selected in "D12 cell" drop-down list. No energy or other cofactors are required in BOF level 1, so sheet Table 2 was left empty. BIOMASS sheet fully completed is illustrated in (Figure 1). A level_2 sheet was created and named "DNA" (Figure 2). *E. Coli* genome GC-content was used to detail DNA composition in sheet Table 1 (24.6% A, 24.6% T, 25.4% C and 25.4% G) using metabolites identifiers (datp_c, dttp_c, dctp_c and dgtp_c) from in iML1515 GEM (Monk et al. 2017). DNA polymerization produces one diphosphate per dNTP addition, therefore production of one mole of ppi_c metabolite per mole of DNA was added in DNA sheet Table 2. Same methodology was applied to create and fill the nine sheets required to generate the BOF matching the structure described above (BIOMASS, DNA, RNA, PROTEINS, LIPIDS, POLYSACCHARIDES, PE, PG and CLPN). In Beck, Hunt et Carlson 2018 polymer lengths for the macromolecular synthesis reactions was set to 10 NTPs for RNA, 10 glucose-1-phosphates for polysaccharides and 100 amino acids for proteins. Polymerization energy requirements and byproducts in Beck, Hunt et Carlson 2018 data are given on the basis of these polymers lengths. On the other hand in BioModTool all polymers are considered to be one monomer long, stoichiometric coefficients of energy and byproducts requirement reported by Beck, Hunt et Carlson 2018 were therefore adjusted.

Fully completed Excel file is available as (Supplemental File S4).

|  | A | B | C | D | E |
| --- | --- | --- | --- | --- | --- |
| 1 | **BIOMASS (level 1) - Instructions** |  |  |  |  |
| 2 | **Please fill the sheet according to the following instructions:** | |  |  |  |
| 3 | **1 - Fill metabolite IDS** | ***Table1 :* Pseudo-metabolite ID (also used for pseudo-reaction ID).  /!\ Add a sheet for each pseudo metabolite. (Sheet name = pseudo-metabolite ID)** | | | |
| 4 |  | *Additional informations can be added.  Names will be added to pseudo-metabolites and pseudo-reactions (optional). Comments/Source cells are not taken into account by BioModTool.* | | | |
| 5 | **2 - Indicate data unit** | ***Table 1:*** Choose appropriate unit  **(in D12 cell).** | Possible units: | **g per …** | for example g/gDCW, g/cells, mass% … |
| 6 |  |  |  | **mol per …** | for example mol/gDCW, mol/cells, mol% … |
| 7 | **3 - Fill coefficients** | ***Table 1:*** coefficient value for each pseudo-metabolite (macromolecule/polymer). **/!\ Coefficient >= 0** | | | |
| 8 |  |  |  |  |  |
| 9 | **BIOMASS (level 1) - Data read by BioModTool** |  |  |  |  |
| 10 | **Table 1: Basic level metabolites**  *coefficient >= 0 Coefficients will be converted and normalized by BioModTool***.** | | | | |
| 11 | **Metabolite name** | **Metabolite ID in model** | **Coefficient** | **Unit** | **Comments/Source** |
| 12 | DNA pseudo-metabolite | **DNA** | 1 | **g per …** | data in gpolymer/gDW  Beck et al., 2018 |
| 13 | RNA pseudo-metabolite | **RNA** | 17.2 | g per … | Beck et al., 2018 |
| 14 | PROTEINS pseudo-metabolite | **PROTEINS** | 35.2 | g per … | Beck et al., 2018 |
| 15 | LIPIDS pseudo-metabolite | **LIPIDS** | 6.7 | g per … | Beck et al., 2018 |
| 16 | POLYSACCHARIDES pseudo-metabolite | **POLYSACCHARIDES** | 4.2 | g per … | Beck et al., 2018 |
| 17 |  |  |  | g per … |  |
| 18 |  |  |  | g per … |  |

**Figure 1. Example of a "BIOMASS" sheet of BioModTool Excel filled with *E. coli* data from Beck et al., 2018.**

|  | A | B | | C | D | E | F |
| --- | --- | --- | --- | --- | --- | --- | --- |
| 1 | **DNA (level 2) - Instructions** | |  |  |  |  |  |
| 2 | **Please fill the sheet according to the following instructions:** | | |  |  |  |  |
| 3 | **1 - Fill metabolite IDS** | | ***Tables 1 & 2: Metabolite IDs.* /!\ Must correspond to a metabolite of the chosen model.** | | | | |
| 4 |  | | *Additional information can be added. Names will be added to pseudo-metabolites and pseudo-reactions (optional). Comments/Source cells are not taken into account by BioModTool.* | | | | |
| 5 | **2 - Indicate data unit** | | ***Table 1:*** Choose appropriate unit | Possible units: | **g per …** | for example g/gDCW, g/cells, mass% … | |
| 6 |  | | **(in D12 cell).** |  | **mol per …** | for example mol/gDCW, mol/cells, mol% … | |
| 7 | **3 - Fill coefficients** | | ***Table 1:*** coefficient value for each pseudo-metabolite (macromolecule/polymer). **/!\ Coefficient >= 0** | | | | |
| 8 |  | |  |  |  |  |  |
| 9 | **DNA (level 2) - Data read by BioModTool** | | |  |  |  |  |
| 10 | **Table 1: Basic level metabolites**  *coefficient >= 0 Coefficients will be converted and normalized by BioModTool***.** | | | | | |  |
| 11 | **Metabolite name** | | **Metabolite ID in model** | **Coefficient** | **Unit** | **Comments/Source** |  |
| 12 | dATP | | **datp_c** | 24.6000 | **mol per …** | data in gpolymer/gDW Beck et al., 2018 |  |
| 13 | dCTP | | **dctp_c** | 25.4000 | mol per … | Beck et al., 2018 |  |
| 14 | dGTP | | **dgtp_c** | 25.4000 | mol per … | Beck et al., 2018 |  |
| 15 | dTTP | | **dttp_c** | 24.6000 | mol per … | Beck et al., 2018 |  |
| 16 |  | |  |  | mol per … |  |  |

Figure 2. Example of a "DNA" sheet of BioModTool Excel filled with *E. coli* data from Beck et al., 2018. *(Continue next page)*

| 1 | **DNA (level 2) - Instructions** |  |  |  |  |
| --- | --- | --- | --- | --- | --- |
| 2 |  |  |  |  |  |
| 3 | ***Tables 1 & 2: Metabolite IDs.* /!\ Must correspond to a metabolite of the chosen model.** | | |  |  |
| 4 | *Additional information can be added. Names will be added to pseudo-metabolites and pseudo-reactions (optional). Comments/Source cells are not taken into account by BioModTool.* | | |  |  |
| 5 | ***Table 2:*** Unit can not be changed (mmol/gDW) | | |  |  |
| 6 |  |  |  |  |  |
| 7 | ***Table 2:*** coefficient value for each metabolite. **/!\ Coefficient < 0 if the metabolite is consumed and >0 if metabolite is produced by the reaction.** | | |  |  |
| 8 |  |  |  |  |  |
| 9 | **DNA (level 2) - Data read by BioModTool** | |  |  |  |
| 10 | **Table 2: Intermediate and advanced levels metabolites (optional)** *if consumed: coeff < 0,  if produced coeff >0 /!\ Coefficients will be directly used in pseudo-reaction (no conversion).* | | | | |
| 11 | **Metabolite name** | **Constant metabolite ID in model** | **Constant metabolite Coefficient** | **Constant metabolite Unit** | **Comments/Source** |
| 12 | Diphosphate | ppi_c | 1.0000 | mol/mol macromolecule | Beck et al., 2018 |
| 13 |  |  |  | mol/mol macromolecule |  |

**End of Figure 2.**

- 1. Run BioModTool

BioModTool can be run either in Python command-lines or using the user interface (implemented with Tkinter).

- - 1. Line-command version

The following section presents an example of a Jupyter Notebook to run BioModTool. This Jupyter Notebook is available: Ecoli_iML1515_script_BioModTool_add_biomass_reaction.ipynb at <https://github.com/Total-RD/BioModTool/tree/main/Application_examples/1_iML1515_ecoli/test_with_jupyternotebook>.

**Addition of a new BOF to iML1515 using BioModTool:**

- Cobra model: iML1515 (*E. coli*) (Monk et al. 2017)
- Biomass composition data from (Beck, Hunt et Carlson 2018)

**Imports**

In [1]:

| import BioModTool.load  import BioModTool.main_add_biomass_objective_function  import BioModTool.save |
| --- |

**1 - Load Genome Scale Metabolic Model**

***1.a) Model directory***

In [2]:

| path_to_model = "path_to_model_repository\\iML1515.xml" |
| --- |

In [3]:

| original_model = BioModTool.load.load_cobra_model(path_to_model) |
| --- |

***1.b) Calculate formula and/or charge ?***

| **/!\ Note that if you select formula = False, the molecular weight cannot be calculated from the metabolite formula preventing unit conversion.**  **- Level 1 data must be given in mmol.gDW-1**  **- Levels 2 and 3 data must be given in mol per ...** |
| --- |

In [4]:

| calculate_formula = True  calculate_charge = True |
| --- |

***1.c) Choose a compartment to add biomass reactions***

- Reactions and pseudo metabolites will be added in compartment given by the user.
- Compartment must be chosen among model's compartments (key of cobra_model.compartments dictionary)
- Biomass reactions are commonly added in cytosol ("_c").

In [5]:

| # Compartments in model:  original_model.compartments |
| --- |

Out[5]:

{'c': 'cytosol', 'e': 'extracellular space', 'p': 'periplasm'}

In [6]:

| BOF_compartment = "c" |
| --- |

**2 – Biomass composition data**

***2.a) Define data file***

Data must be given in an Excel file with a specific format. See Supplemental Files S1-S3.

In [7]:

| path_to_data = "Biomass_composition_ecoli_Beck2018.xlsx" |
| --- |

**3 – Structure of biomass objective function**

***3.a) Biomass reaction structure***

Dictionary defining BOF structure:

Must be defined in accordance with Excel data file (sheet names).

- Mandatory:
  - one and only one key with value = "level_1"
- Optional:
  - if desired structure contains two or more levels:
    - one or several key(s) with value = "level_2"
  - if desired structure contains three levels:
    - one and only one key with value = "level_2_lipid"
    - one or several key(s) with value = "level_3"

In [8]:

| dict_pool_id_Ecoli = {'BIOMASS': 'level_1',  'POLYSACCHARIDES': 'level_2',  'DNA': 'level_2',  'RNA': 'level_2',  'PROTEINS': 'level_2',  'LIPIDS': 'level_2_lipid',  'PG': "level_3",  'PE': "level_3",  'CLPN': "level_3"} |
| --- |

***3.b) Choose a suffix***

- Expected suffix: string containing only alphanumeric characters or _
- Test performed in BioModTool: re.match("^[a-zA-Z0-9_]*$",suffix)
- All added pseudo-reactions and pseudo-metabolites will contain the given suffix.

In [9]:

| suffix = "BioModTool_beck2018" |
| --- |

**4 – Create and add the new biomass objective function**

In [10]:

| updated_model = BioModTool.main_add_biomass_objective_function.add_biomass_objective_function(  cobra_model = original_model,  path_to_data = path_to_data,  suffix = suffix,  dict_structure = dict_structure_BOF,  user_compartment = BOF_compartment,  calculate_charge = calculate_charge,  calculate_formula = calculate_charge,  saving_final_data = True) |
| --- |

Out[10]:

Metabolite POLYSACCHARIDES_BioModTool_beck2018_c (formula: C6.0H10.0O5.0, charge: 0) added to model.

Reaction POLYSACCHARIDES_BioModTool_beck2018_c added to model.

atp_c + g1p_c --> POLYSACCHARIDES_BioModTool_beck2018_c + adp_c + ppi_c

------------------------------------------------------------------------------

Metabolite DNA_BioModTool_beck2018_c (formula: C9.746H11.246O6.0N3.754P1.0, charge: -1) added to model.

Reaction DNA_BioModTool_beck2018_c added to model.

0.246 datp_c + 0.254 dctp_c + 0.254 dgtp_c + 0.246 dttp_c --> DNA_BioModTool_beck2018_c + ppi_c

------------------------------------------------------------------------------

[…]

------------------------------------------------------------------------------

Metabolite BIOMASS_BioModTool_beck2018_c (formula: C40.59259727004123H62.339155478773726O16.850976170654622N10.09717316334253P1.037995606777102S0.2007301352, charge: -1) added to model.

Reaction BIOMASS_BioModTool_beck2018_c added to model.

0.0505 DNA_BioModTool_beck2018_c + 0.13999 LIPIDS_BioModTool_beck2018_c + 0.40285 POLYSACCHARIDES_BioModTool_beck2018_c + 5.02831 PROTEINS_BioModTool_beck2018_c + 0.84039 RNA_BioModTool_beck2018_c --> BIOMASS_BioModTool_beck2018_c

------------------------------------------------------------------------------

**5 – Save updated model**

BioModTool comes with a function to save model both in JSON and SBML formats.

In [11]:

| BioModTool.save.save_model(updated_model,"iML1515_updated" ) |
| --- |

- - 1. Using graphical interface

BioModTool comes with a graphical user interface: interface_BioModTool.py. Interface source code is available on GitHub (<https://github.com/Total-RD/BioModTool/blob/main/interface_BioModTool.py>). The interface can be executed using Python and is compatible with Windows, Linux and MacOS operating systems.

*python <path-to-interface_BioModTool.py>*

On Windows, the interface can also be launched directly from an executable: interface_BioModTool.exe

N.B.: interface_BioModTool.exe is in the repository **BioModTool\dist**

**Step 1:** Browse GEM model file (JSON or SBML format) and select via radio buttons if formula and/or charge are available for all metabolites consumed in the BOF.


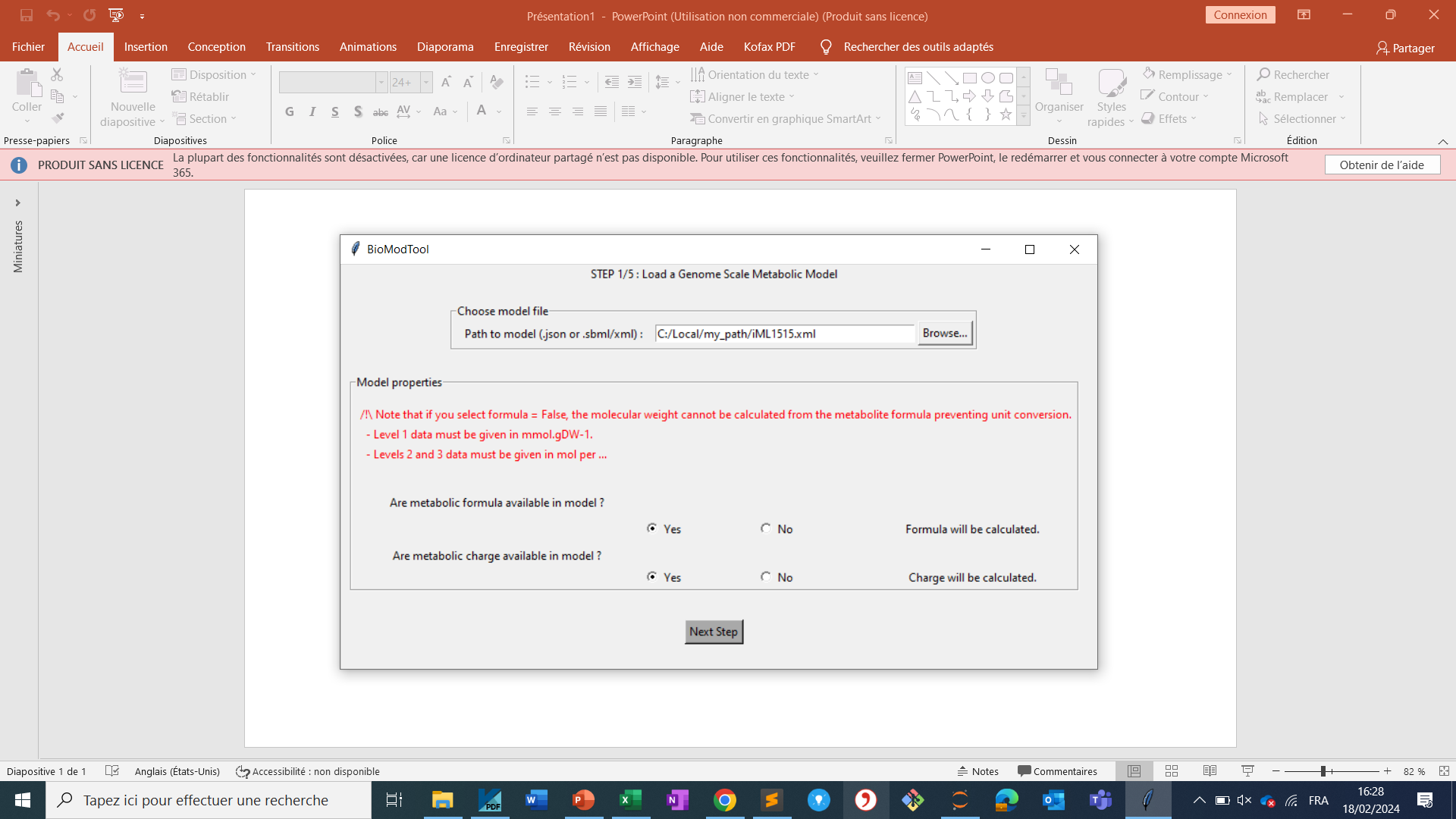


**Step 2:** Select a compartment in the drop-down list. Biomass objective function will be added to the selected compartment.


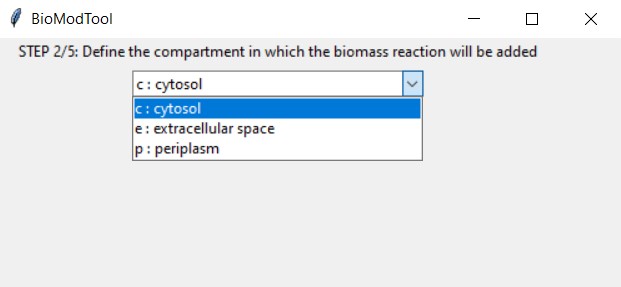


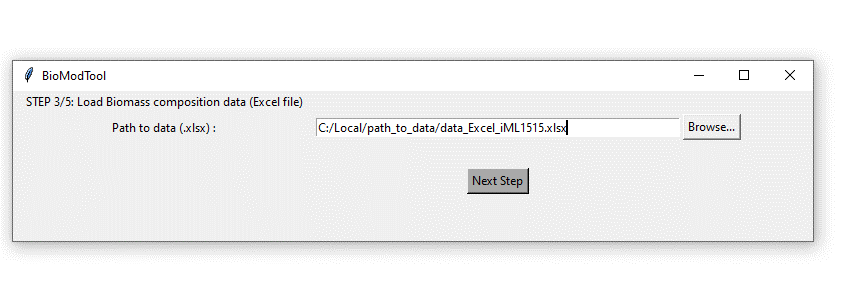
**Step 3:** Browse biomass composition data (Excel file, *.xlsx).

**Step 4:** Based on Excel data file structure (available sheets), define BOF structure. To do so, indicate desired level for each pseudo-metabolite by selecting desired level: level_1, level_2, level_2_lipide or level_3.


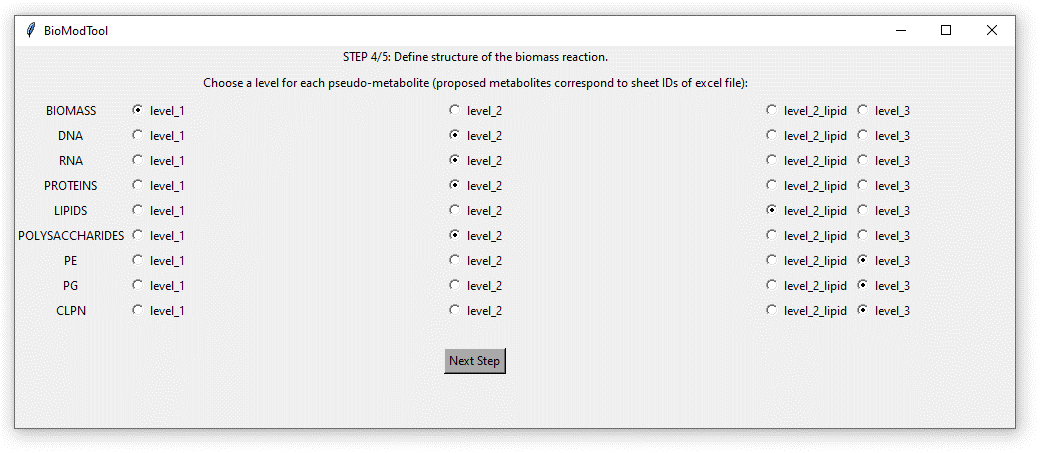


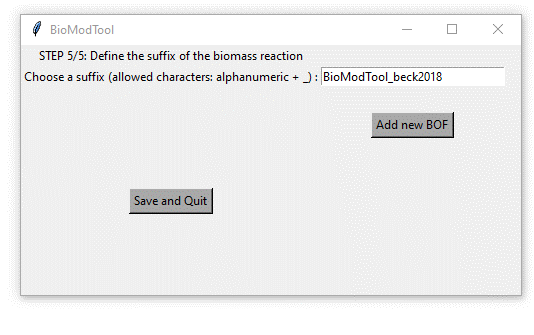
**Step 5:** Define a suffix, only alphanumeric characters (and "_") are accepted. Add the BOF, save and quit BioModTool.

Updated model is saved in SBML and JSON formats in a directory chosen by the user. Excel file with calculations details is saved in the working directory.

- 1. Remove a BOF with BioModTool

A function removing a BioModTool formatted BOF from GEM is available (remove_biomass_objective_function). The function takes a model and a suffix as parameters, remove all reactions harboring the given suffix ("_suffix_") in their ID and returns updated model.

This functionality is available in line command version.

In [12]:

| model_wo_BOF = remove_biomass_objective_function(updated_model,suffix) |
| --- |

1. Implementation

First, BioModTool loads and tests user inputs. Then, stoichiometric coefficients are calculated from data.

For levels 2 and 3 pseudo-reactions, final stoichiometric coefficients are in molar fraction (mol.mol^-1^). For each pseudo-reaction if user data are in "g per …", data are converted in "mol per …" as follows:

$${coeff}_{mol}= \frac{{coeff}_{g}}{MW}$$

With ${coeff}_{mol}$ molar coefficient in "mol per …", ${coeff}_{g}$ initial mass coefficient in "g per …" and $MW$ molecular weight of the metabolite calculated from metabolite formula in the GEM.

In BioModTool, $MW$ are calculated using COBRApy function *formula_weight* from metabolite instances (Ebrahim et al. 2013). Molar coefficients are then normalized so that the sum of all molar fractions is one, and rounded to the fifth decimal place.

By definition, produced biomass must represent one gram of dry weight. Therefore, for level 1 pseudo-reaction (typically biomass), final stoichiometric coefficients are in mmol.gDW^-1^. Before being converted in mmol.gDW^-1^, coefficients given by the user are first converted in g.gDW^-1^ to allow their normalization. Data in "mol per …" are converted in "g per …" as follows:

$${coeff}_{g}= {coeff}_{mol}* MW$$

With ${coeff}_{g}$ mass coefficient in "g per …", ${coeff}_{mol}$ initial molar coefficient in "mol per …" and $MW$ molecular weight of the metabolite obtained from metabolite formula. Mass coefficients are then normalized such that the sum of metabolite mass fraction is one, before being converted in final unit mmol.gDW^-1^ as follows:

$${coeff}_{mmol/gDW}= 1000* \frac{{coeff}_{g/gDW}}{MW}$$

With ${coeff}_{mmol/gDW}$ final stoichiometric coefficient in mmol.gDW^-1^, ${coeff}_{g/gDW}$ mass fraction in g.gDW^-1^ and $MW$ molecular weight of the metabolite. Final coefficients are rounded to fifth decimal place. BioModTool calculates pseudo-metabolites charge and formula (when possible and desired by the user), and instantiates pseudo-metabolites and pseudo-reactions. Annotation being an important feature in GEM models, SBO terms "SBO:0000247" and "SBO:0000629" are respectively added to metabolites and reactions annotations (Lieven et al. 2020).

In accordance with the FAIR (Findable, Accessible, Interoperable, Reusable) principles and to offer users a comprehensible overview of the calculations executed by BioModTool, the results of the various calculations steps are recorded in an Excel file. The pseudo-metabolites created, along with their charge and chemical formula, are also summarized in this Excel file (worksheet: added_metabolites). In addition, BioModTool performs a charge and mass balance test of all the reactions making up the biomass function. Results of this test are saved in the Excel file (worksheet: mass_charge_balance).

Steps that require loading, updating, saving GEM and manipulating metabolites and reactions rely on COBRApy packages (Ebrahim et al. 2013). Full codes are available at (<https://github.com/Total-RD/BioModTool>).

References

Beck, Ashley; Hunt, Kristopher; Carlson, Ross (2018): Measuring Cellular Biomass Composition for Computational Biology Applications. In Processes 6 (5), p. 38. DOI: 10.3390/pr6050038.

Ebrahim, Ali; Lerman, Joshua A.; Palsson, Bernhard O.; Hyduke, Daniel R. (2013): COBRApy: COnstraints-Based Reconstruction and Analysis for Python. In BMC Syst Biol 7 (1), p. 74. DOI: 10.1186/1752-0509-7-74.

Feist, Adam M.; Palsson, Bernhard O. (2010) The biomass objective function. In Current opinion in microbiology, vol. 13, n° 3, p. 344–349. DOI: 10.1016/j.mib.2010.03.003.

Lachance, Jean-Christophe; Lloyd, Colton J.; Monk, Jonathan M.; Yang, Laurence; Sastry, Anand V.; Seif, Yara et al. (2019): BOFdat: Generating biomass objective functions for genome-scale metabolic models from experimental data. In PLoS computational biology 15 (4), e1006971. DOI: 10.1371/journal.pcbi.1006971.

Lieven, Christian; Beber, Moritz E.; Olivier, Brett G.; Bergmann, Frank T.; Ataman, Meric; Babaei, Parizad et al. (2020): MEMOTE for standardized genome-scale metabolic model testing. In Nature biotechnology 38 (3), pp. 272–276. DOI: 10.1038/s41587-020-0446-y.

Monk, Jonathan M.; Lloyd, Colton J.; Brunk, Elizabeth; Mih, Nathan; Sastry, Anand; King, Zachary et al. (2017): iML1515, a knowledgebase that computes Escherichia coli traits. In Nature biotechnology 35 (10), pp. 904–908. DOI: 10.1038/nbt.3956.

Simensen, Vetle; Schulz, Christian; Karlsen, Emil; Bråtelund, Signe; Burgos, Idun; Thorfinnsdottir, Lilja Brekke et al. (2022) Experimental determination of Escherichia coli biomass composition for constraint-based metabolic modeling. In PloS one 17 (1) e0262450. DOI: 10.1371/journal.pone.0262450.

Thiele, Ines; Palsson, Bernhard Ø. (2010). A protocol for generating a high-quality genome-scale metabolic reconstruction. In Nature protocols 5 (1), p. 93–121. DOI: 10.1038/nprot.2009.203.

Tolleter, Dimitri; Smith, Edward N.; Dupont-Thibert, Clémence; Uwizeye, Clarisse; Vile, Denis; Gloaguen, Pauline et al. (2024) The Arabidopsis leaf quantitative atlas: a cellular and subcellular mapping through unified data integration. In Quant Plant Biology 5. DOI: 10.1017/qpb.2024.1.
